# Impact of COVID-19 on health-related quality of life in patients with cardiovascular disease: a multi-ethnic Asian study

**DOI:** 10.1186/s12955-020-01640-5

**Published:** 2020-12-14

**Authors:** Shir Lynn Lim, Kai Lee Woo, Eleanor Lim, Faclin Ng, Mark Y. Chan, Mihir Gandhi

**Affiliations:** 1 Department of Cardiology, National University Heart Center, Singapore, Singapore; 2 Department of Medicine, Yong Loo Lin School of Medicine, Singapore, Singapore; 3 Biostatistics, Singapore Clinical Research Institute, Singapore, Singapore; 4 Center for Quantitative Medicine, Duke-NUS Medical School, Singapore, Singapore; 5 Global Health Group, Center for Child Health Research, Tampere University, Tampere, Finland

**Keywords:** COVID-19, Health-related quality of life, Cardiovascular, EQ-5D, Psychological health

## Abstract

**Background:**

Little is known about the impact of the global coronavirus disease-2019 (COVID-19) pandemic on patients with cardiovascular disease (CVD), the biggest global killer and major risk factor for severe COVID-19 infections. We aim to explore the indirect consequences of COVID-19 on health-related quality of life (HRQoL) of patients with CVD.

**Methods:**

Eighty-one adult outpatients with CVD were assessed using the EQ-5D, a generic health status instrument with five dimensions (mobility, self-care, usual activities, pain/discomfort, anxiety/depression), before and during the pandemic. Changes in the EQ-5D dimensional responses were compared categorically as well as using the dimension-specific sum-score (range 1–3, with a higher score indicating worse health). The responses and sum-score were compared using the exact test of symmetry and the paired *t*-test, respectively.

**Results:**

These patients [mean age (SD) 59.8 (10.5); 92.6% males; 56% New York Heart Association (NYHA) functional class I] had coronary artery disease (69%), heart failure (28%), or arrhythmias (15%). None experienced change in NYHA class between assessments. About 30% and 38% of patients reported problems with at least one of the EQ-5D dimensions pre-pandemic and during the pandemic, respectively. The highest increase in health problems was reported for anxiety/depression (12.5% pre-pandemic vs 23.5% during pandemic; *p* = 0.035) with mean domain-specific score from 1.12 (SD 0.33) to 1.25 (SD 0.46) (standardized effect size = 0.373, *p* = 0.012). There was no meaningful change in other dimensions as well as overall HRQoL.

**Conclusion:**

The COVID-19 pandemic is associated with a significant worsening of the mental health of patients with CVD.

## Introduction

Coronavirus disease 2019 (COVID-19) is a global pandemic with unprecedented medical, economic and social consequences. Much attention has been on the potentially severe cardiovascular complications associated with COVID-19 [1, 2]; patients with pre-existing cardiovascular disease (CVD) also suffer worse outcomes with COVID-19 infection [3]. Beyond these direct consequences, COVID-19 has reshaped the delivery of cardiovascular care—non-critical and elective procedures are postponed, and distancing imperatives have led to rapid scaling of telemedicine and cancellation of cardiac rehabilitation. The repeated public health messages that those with chronic conditions should practice social and physical distancing may adversely affect the health-seeking behavior of patients with CVD. Indeed, data from affected countries show reductions in hospital admission for myocardial infarctions and strokes [4], but those who presented were sicker and had worse outcomes, suggesting delayed presentation [5, 6].

Studies evaluating the impact of COVID-19 on non-infected persons have also looked at the psychological impact of the pandemic [7, 8]; both small and large studies have been conducted in general population, healthcare workers, and those with pre-existing psychiatric diseases. Little is known about the impact of COVID-19 on physical and mental health of patients with CVD, the biggest killer globally [9].

Singapore is a multi-ethnic city state in the Asia–Pacific; majority of its 5.7 million population are Chinese, followed by Malays and Indians. It reported its first case of COVID-19 on 23rd January 2020, and imposed a partial national lockdown, termed as the Circuit Breaker, from 3rd April 2020 [10]. There was discontinuation of non-critical medical services, mask advisory and closure of public places. There was gradual easing of the restrictions from 2nd June 2020 (Phase 1 Safe Re-opening) [11], followed by Phase 2 Re-opening from 18th June 2020, where almost all activities were allowed except for large-scale events and venues, as well as entertainment venues [12].

Our study aimed to, using the EQ-5D, evaluate the impact of COVID-19 on health-related quality of life (HRQoL) in a multi-ethnic Asian cohort with CVD in Singapore, hypothesizing that the physical capacity and mental health of these patients would be adversely affected by COVID-19.

## Methods

### Study design and setting

This was a longitudinal survey involving a multi-ethnic Asian cohort of adult outpatients with known CVD in National University Hospital, Singapore, a 1200-bed tertiary hospital and a major referral center. Recruitment took place between 29th April to 19th June 2020, commencing approximately 4 weeks after partial lockdown was imposed, till the end of Phase 1 Safe Re-opening.

### Study population

Asian patients with known CVD were eligible if they were at least 21 years of age, had completed a HRQoL questionnaire prior to the COVID-19 outbreak, and agreed to participate. Asian was defined as a person having origins in any of the original peoples of the Far East, Southeast Asia, or the Indian subcontinent, for example, Cambodia, China, India, Japan, Korea, Malaysia, Singapore, Pakistan, Philippines, Thailand and Vietnam [13]. The diagnosis of CVD (coronary artery disease, heart failure, arrhythmias, others) was based on internationally accepted criteria, and performed by their managing cardiologists. In addition, these patients were part of previous research projects which collected data on HRQoL [Survey on Heart Disease Patients’ Health-related Quality of Life and Preferences (CIRB Reference: 2017/2125), Improving Outcomes in Acute Myocardial Infarction Through Reversal of Early and Late Cardiac Remodelling Study (DSRB Ref: 2014/00793) and Nitrates In Combination with Hydralazine in Cardiorenal Syndrome Study (DSRB Ref: 2014/00790)] and were referred by their managing cardiologists for participation during outpatient consultations. Patients were recruited consecutively with no restrictions on type and stage of CVD.

Patients were excluded if they were illiterate or refused to provide consent.

Verbal consent for participation was obtained over the phone, prior to follow-up survey. This study was approved by the Domain Specific Review Board (NHG DSRB Ref: 2020/00436) and conducted according to the World Medical Association Declaration of Helsinki.

### Assessments

Our study used EQ-5D questionnaires for HRQoL assessments. The EQ-5D is one of the most commonly used generic preference-based health status questionnaires [14]. Three-level EQ-5D (EQ-5D-3L) contains five dimensions: mobility, self-care, usual activities, pain/discomfort, and anxiety/depression, each with three response levels (no problems, some/moderate problems or severe/extreme problems). It also has a visual analog scale (EQ-VAS) measuring health on the scale of 0 to 100, with a higher score indicating better health. A five-response level version was later developed (EQ-5D-5L) to enhance its sensitivity and reduce ceiling effect [15]. Both the versions have been psychometrically validated in a large number of diseases and conditions, including cardiovascular health [16].

Our study used official English and Chinese versions of EQ-5D-3L and EQ-5D-5L questionnaires. The utility scores for EQ-5D-3L and EQ-5D-5L were derived from the Singapore value set for EQ-5D-3L and its crosswalk algorithm, respectively [17]. Utility scores range from − 0.769 to 1, with higher scores indicating better health. Dimension-specific sum score was calculated as the mean of each dimension-specific item responses. Dimension-specific score range from 1 to 3 for EQ-5D-3L and 1 to 5 for EQ-5D-5L, with a higher score indicating worse health.

The EQ-5D-3L and EQ-VAS were administered to all study participants. In addition, a subset of participants also administered the EQ-5D-5L. EQ-5D-3L and EQ-5D-5L were administered in random order to avoid sequence effect on the responses. The pre-pandemic questionnaires were self-administered by the participants during physical outpatient visits. However, due to restrictions in face-to-face visits during the pandemic, the follow-up assessments were conducted remotely, either via the phone or through the mail.

In addition to EQ-5D questionnaires, the study collected sociodemographic and clinical information on patient CVD diagnosis, comorbidities and New York Heart Association (NYHA) functional class. Clinical information was recorded from patients’ medical records available at the hospital. NYHA functional class was assessed during both pre- and during pandemic visits.

### Sample size

We anticipated that pandemic might have a mild-to-moderate impact on patients’ HRQoL. The sample size for the study was estimated to detect a change of one-third of standard deviation (SD) (paired difference divided by SD = 0.33 standardized effect size) based on dimension-specific scores [18]. A sample size of 75 participants was required to detect the desired change in a dimension-specific score of 0.33 standardized effect size with 80% power at a 5% two-sided level of significance using the paired *t*-test [19]. The sample size was not adjusted for multiplicity due to the exploratory nature of this study. The sample size was calculated using PASS 2020 Power Analysis and Sample Size Software (2020). NCSS, LLC, US.

### Statistical analysis

The mean (SD) of pre-pandemic and pandemic, as well as change in dimension-specific scores were calculated for all EQ-5D dimensions. Two-sided paired *t*-tests were used to assess the statistical significance of the change in the scores. The changes in scores were also presented with the standardized effect size (SES) estimated as the mean paired difference of during and pre-pandemic assessments divided by the standard deviation of pre-pandemic assessment. SES was considered small (0.2), medium (0.5), and large (0.8) according to Cohen’s recommendation [18].

Dimension-level item responsiveness was evaluated using a percentage of participants in each of the severity level at pre- and during-pandemic assessments. The distribution of participants between the two assessments was compared using the McNemar–Bowker exact test of symmetry.

EQ-VAS and utility scores for EQ-5D-3L and EQ-5D-5L were analyzed similarly to dimension-specific scores.

The analysis based on the EQ-5D-3L and EQ-5D-5L data were considered as the primary analysis and sensitivity analyses, respectively, as EQ-5D-5L was administered to only a subset of participants. Sensitivity analyses were also performed on the EQ-5D-3L and EQ-5D-5L data using the Wilcoxon signed-rank test to evaluate the robustness of the paired *t*-test results.

## Results

A total of 85 patients with pre-pandemic HRQoL assessments were approached between 29th April 2020 to 19th June 2020 for this study, and 84 (99%) agreed to participate. Data from 3 (4%) participants were excluded from the final analysis, as the baseline HRQoL survey was performed more than 2 years ago. The final number of participants whose data were analyzed was 81.

The mean age of the participants was 60 years (SD 11), the majority were men (93%), and the ethnic distribution reflected that of the general population. The majority of the participants had coronary artery disease (69%), followed by chronic heart failure (28%). Along with their CVD, the participants also reported having other comorbidities such as hypertension (62%), dyslipidemia (61%), and diabetes mellitus (49%). There was little functional limitation at baseline with 94% reporting NYHA functional classes I or II. There was no change in NYHA functional status during the pre- and during-pandemic visits for any of the participants with 97% reporting NYHA functional classes I or II during pandemic. The baseline characteristics of the patients are summarized in Table 1.

**Table 1:** Patient characteristics at pre-pandemic visit

Characteristics	N = 81
Age (years), mean (SD)	59.8 (10.5)
Male, n (%)	75 (92.6)
Ethnicity, n (%)	
Chinese	62 (76.5)
Malay	12 (14.8)
Indian	6 (7.4)
Others	1 (1.2)
Education, n (%)	
Primary (6 years) or less	13 (16.1)
Secondary (up to 11 years)	35 (43.2)
Diploma, university or higher	33 (40.7)
Household earning < S$4000, n (%)	42 (51.9)
Heart problems, n (%)	
Coronary artery disease	56 (69.1)
Heart failure	23 (28.4)
Arrhythmia	12 (14.8)
Other heart problems	6 (7.4)
Comorbidities, n (%)	
Hypertension	50 (61.7)
Hyperlipidaemia	49 (60.5)
Diabetes	40 (49.4)
Stroke	9 (11.1)
Other*	40 (49.4)
NYHA functional classification, n (%)	
I	45 (55.6)
II	31 (38.3)
III	5 (6.2)
Health status using EQ-VAS, mean (SD)	78.6 (12.6)
EQ-5D-3L utility score, mean (SD)	0.898 (0.20)

^*^Includes one or more of the following: chronic lung disease, chronic kidney disease, peripheral vascular disease, cancer, depression/anxiety, chronic liver disease, orthopaedic disorders

*SD* standard deviation, *NYHA* New York Heart Association, *EQ-VAS* EQ-Visual Analogue Scale, *EQ-5D-3L* 3-level EQ-5D

Among 81 patients who participated in the study, all completed EQ-5D-3L at both visits. A subset of 66 patients completed EQ-5D-5L in addition to EQ-5D-3L at both visits. There were no notable differences in baseline characteristics between patients who completed both questionnaires versus who completed only EQ-5D-3L, except for NYHA status and CVD diagnoses (Additional file 1: Table S1).

**Fig. 1:**
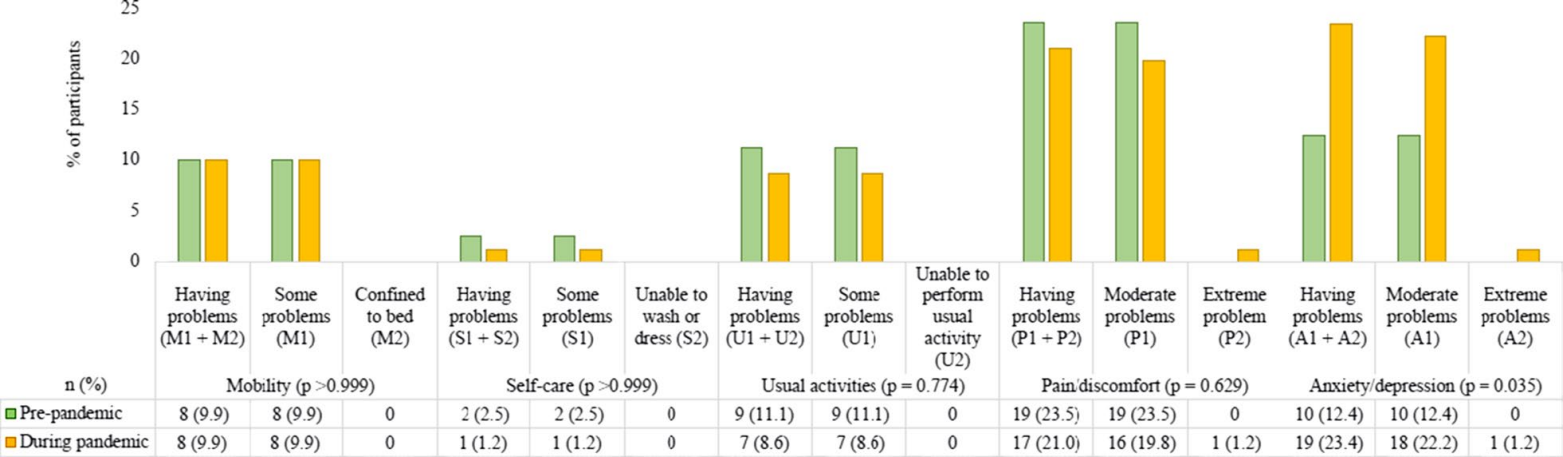
Comparison of EQ-5D-3L responses at pre- and during pandemic visits. Response levels were compared between two visits using the McNemar–Bowker exact test of symmetry. *EQ-5D-3L* 3-level EQ-5D

Using EQ-5D-3L at pre-pandemic, 24 of 81 participants (29.6%) reported problems in at least one dimension; the most commonly affected dimension was pain/discomfort (23.5%) followed by anxiety/depression (12.4%). During the pandemic, 31 participants (38.3%) reported problems in at least one dimension with the severity of the problems in each dimension similar to or worse than pre-pandemic (Fig. 1). Among all the dimensions, the highest increase in problems was reported for anxiety/depression with mean domain-specific score increased by 0.12 (SES = 0.373; *p* = 0.012) (Table 2). There was no meaningful change in EQ-VAS (mean change = − 0.01; 95% CI − 2.87 to 2.85) as well as EQ-5D-3L utility score (mean change = − 0.015; 95% CI − 0.063 to 0.034).

**Table 2:** Comparison of EQ-5D-3L dimension scores at pre- and during-pandemic visits

Dimension	Pre-pandemic	During pandemic	*p *value
Mobility			
Mean score (SD)	1.099 (0.30)	1.099 (0.30)	
Mean change (95% CI)	0.000 (− 0.078, 0.078)		> 0.999
Standardized effect size	0.000		
Self-care			
Mean score (SD)	1.025 (0.16)	1.012 (0.11)	
Mean change (95% CI)	− 0.012 (− 0.055, 0.030)	0.567	
Standardized effect size	0.075		
Usual activities			
Mean score (SD)	1.111 (0.32)	1.086 (0.28)	
Mean change (95% CI)	− 0.025 (− 0.110, 0.061)		0.567
Standardized effect size	0.078		
Pain/discomfort			
Mean score (SD)	1.235 (0.43)	1.222 (0.45)	
Mean change score (95% CI)	− 0.012 (− 0.126, 0.101)		0.829
Standardized effect size	0.028		
Anxiety/depression			
Mean score (SD)	1.123 (0.33)	1.247 (0.46)	
Mean change (95% CI)	0.123 (0.028, 0.218)		0.012
Standardized effect size	0.373		

Dimension score was compared between two visits using the paired *t*-test

*EQ-5D-3L* 3-level EQ-5D, *SD* standard deviation, *CI* confidence interval

A higher proportion of patients reported problems using the EQ-5D-5L compared to the EQ-5D-3L both at pre-pandemic and during pandemic visits. A total 32 of 66 participants (48.5%) reported problems in at least one dimension at pre-pandemic with the majority reported problems in pain/discomfort (39.4%), followed by anxiety/depression (25.8%). During the pandemic, 37 participants (56.1%) reported problems in at least one dimension with the severity of the problems in each dimension similar to or worse than pre-pandemic. Similar to EQ-5D-3L data, the highest increase in problems was reported for anxiety/depression with mean domain-specific score increased by 0.26 (SES = 0.516; *p* = 0.007) (Additional file 1: Table S2). There was no meaningful change in the EQ-5D-5L utility score (mean change = − 0.036; 95% CI − 0.084 to 0.012).

Sensitivity analyses based on the Wilcoxon signed-rank test gave similar results as the paired *t*-test for all the outcomes based on the EQ-5D-3L and EQ-5D-5L (results not shown).

## Discussion

Our study involving patients with chronic CVD reported worse HRQoL in several aspects of health during the COVID-19 outbreak, despite stability in their CVD. Specifically, there was increased anxiety and depression on serial EQ-5D-3L assessments. Results were more pronounced using EQ-5D-5L. The increase in anxiety and depression during the outbreak was clinically (small-to-moderate effect size) as well as statistically significant. To our knowledge, this is one of the first studies evaluating the impact of COVID-19 pandemic on the HRQoL of non-infected persons with CVDs.

Infectious diseases outbreaks are known to have health, social and economic implications, for infected and non-infected persons alike. Prior studies have shown the adverse psychological impact of COVID-19 and its containment measures in COVID-19 patients, frontline workers, patients with pre-existing psychiatric diseases and the general population [7, 8]. Our findings are consistent with prior literature, and further extended those reports by providing the perspectives of those with CVD.

The increase in anxiety and depression amongst our study participants, is unsurprising and may be explained by the following: (1) the swathe of information, from official organizations to social media platforms, re-iterating the increased risks of COVID-19 with CVDs, (2) postponements of non-critical CVD follow-up appointments, and the emphasis on social and physical distancing may have altered their perception of healthcare accessibility, (3) separation from family and friends during the Circuit Breaker, leading to social isolation, (4) mask-wearing requirement in public areas, resulting in dyspnea for some people, and (5) economic concerns, given the closures of businesses. Lingering memories of the devastating impact of the Severe Acute Respiratory Syndrome (SARS) outbreak in 2003, with 238 cases and 33 deaths in Singapore, may have also contributed to anxiety and depression [20]. An understanding of the relative contribution of these factors would have been helpful in shaping future policies and interventions; unfortunately, such data were not collected in this brief study.

The magnitude of change in HRQoL was small. In particular, there was little change in the physical dimensions of HRQoL, which partly reflected the stability of the participants’ underlying CVDs between the surveys done at baseline and during the COVID-19 outbreak. The subgroups bearing the brunt of the pandemic and its containment measures are usually the elderly [21], females [22–24], less educated [24, 25], and those from the low socioeconomic status [26, 27]. Our study participants of mostly older, middle-class males may not have been the vulnerable group. Furthermore, support from the government in response to the pandemic—rapid ramping up of resources and capabilities in terms of COVID-19 testing and provision of care, assurance that non-COVID care would not be compromised, shift towards telemedicine, and massive stimulus packages to cushion the economic impact, might have somewhat mitigated the impact of the pandemic.

There are several strengths of our study. First, all the HRQoL assessments were completed by participants themselves rather than proxies. Second, all participants had baseline assessments prior to the outbreak, thus acting as their own controls. Third, the impact of the pandemic on HRQoL was evaluated by two validated HRQoL measures (EQ-5D-3L and EQ-5D-5L) and both gave consistent results, leaving less room for random noise in the results. Fourth, the follow-up HRQoL assessment was carried out during the partial national lockdown which enabled us to adequately capture the impact of outbreak and containment measures on HRQoL.

Our study has a few limitations. First, the administration of HRQoL assessments was different pre-pandemic and during pandemic for some participants. All pre-pandemic HRQoL assessments were self-administered; majority of the follow-up assessments were administered by the interviewer over the phone. The follow-up assessments were conducted when physical outpatient visits were restricted. Although our preference was for self-administered assessments via email or post, some participants preferred interviewer-administered assessments over the phone. Therefore, we would not rule out some impact of this administration on their responses (response bias). Nevertheless, previous research has shown that interviewer-administration is likely to overestimate HRQoL, rather than underestimate [28]. That is, some participants could have under-reported their health problems. Therefore, our estimates of the impact of the pandemic on HRQoL should be considered as conservative estimates. Second, lack of data on external factors (social, environmental and economic factors) and limited sample size precluded us from studying the potential impact of patient and external factors on the change in HRQoL with certainty. Furthermore, our study performed several hypotheses, including evaluating the pandemic impact on each dimension of EQ-5D separately, without multiplicity correction. There could be chances of inflated type-I error (false positive), and results should be considered as exploratory. Third, as in all survey-based studies, sampling bias is an inherent limitation. Although consecutive recruitment without restrictions on type and stage of CVD allowed for a representative sample of adult patients with CVD, we included only those who were literate (English and Mandarin) and who had completed HRQoL questionnaires prior to the pandemic, thereby selecting the subgroups who may not have been most affected by the pandemic. It is conceivable that the mental health impact could be greater on the subgroups not represented in this study. Finally, our study was conducted in Asian patients with CVD. The findings may not be generalizable to other ethnicities or patient populations. We encourage further research in other countries and other disease groups to understand the impact of the pandemic.

The findings of adverse psychological impact on patients with CVD have important implications. It is plausible that the impact would be greater if our findings are extrapolated to more vulnerable populations—elderly, females, less educated, lower socioeconomic classes and poorly controlled CVD. Psychological ramifications can be long-lasting even after the pandemic has ended, and should not be ignored. At time of writing, there are no authoritative organizations that plan and coordinate psychological interventions in Singapore during the COVID-19 outbreak. In the meantime, cardiologists can do their part by proactively screening for psychological issues in patients who come for consultations, be it in-person or via telemedicine. Where necessary, psychosocial interventions can be implemented in collaboration with psychiatrists and psychologists.

## Conclusion

The COVID-19 pandemic and its containment measures is associated with a significant decline in the psychological health components of the HRQoL of patients with pre-existing CVD. Our data suggest a need for improved screening of psychological issues and timely implementation of psychosocial interventions in these patients during the pandemic.

## Supplementary Information

**Additional file 1.** Supplemental Tables S1 and S2.

## Data Availability

The dataset used and analyzed during the current study are available from the corresponding author on reasonable request.

## Abbreviations

COVID-19: Coronavirus disease 2019
CVD: Cardiovascular disease
HRQoL: Health-related quality of life
EQ-5D-3L: Three-level EQ-5D
EQ-VAS: EQ visual analogue scale
EQ-5D-5L: Five-level EQ-5D
NYHA: New York Heart Association
SD: Standard deviation
SES: Standardized effect size
SARS: Severe Acute Respiratory Syndrome

## References

[1] Fried JA, Ramasubbu K, Bhatt R (2020). The variety of cardiovascular presentations of COVID-19. Circulation.

[2] Clerkin KJ, Fried JA, Raikhelkar J (2020). COVID-19 and cardiovascular disease. Circulation.

[3] Wu Z, McGoogan JM (2020). Characteristics of and important lessons from the coronavirus disease 2019 (COVID-19) outbreak in China: summary of a report of 72 314 cases from the Chinese Center for Disease Control and Prevention. JAMA.

[4] Garcia S, Albaghdadi MS, Meraj PM (2020). Reduction in ST-segment elevation cardiac catheterization laboratory activations in the United States during COVID-19 pandemic. J Am Coll Cardiol.

[5] Baldi E, Sechi GM, Mare C (2020). Out-of-hospital cardiac arrest during the Covid-19 outbreak in Italy. N Engl J Med.

[6] Marijon E, Karam N, Jost D (2020). Out-of-hospital cardiac arrest during the COVID-19 pandemic in Paris, France: a population-based, observational study. Lancet Public Health.

[7] Vindegaard N, Benros ME (2020). COVID-19 pandemic and mental health consequences: systematic review of the current evidence. Brain Behav Immun.

[8] Rajkumar RP (2020). COVID-19 and mental health: a review of the existing literature. Asian J Psychiatry.

[9] World Health Organization. Cardiovascular diseases (CVDs). 2017. https://www.who.int/news-room/fact-sheets/detail/cardiovascular-diseases-(cvds). Accessed 10 July 2020.

[10] Ministry of Health. Updates on COVID-19 (Coronavirus Disease 2019) local situation. Circuit breaker to minimise further spread of COVID-19. 2020. https://www.moh.gov.sg/news-highlights/details/circuit-breaker-to-minimise-further-spread-of-covid-19. Accessed 10 July 2020.

[11] Ministry of Health. Updates on COVID-19 (Coronavirus Disease 2019) local situation. End of circuit breaker, phased approach to resuming activities safely. 2020. https://www.moh.gov.sg/news-highlights/details/end-of-circuit-breaker-phased-approach-to-resuming-activities-safely. Accessed 10 July 2020.

[12] Ministry of Health. Updates on COVID-19 (Coronavirus Disease 2019) local situation. Moving into phase two of re-opening. 2020. https://www.moh.gov.sg/news-highlights/details/moving-into-phase-two-of-re-opening. Accessed 10 July 2020.

[13] US Census Bureau. 2020. https://www.census.gov/topics/population/race/about.html. Accessed 10 Nov 2020.

[14] Rabin R, de Charro F (2001). EQ-5D: a measure of health status from the EuroQol Group. Ann Med.

[15] Herdman M, Gudex C, Lloyd A (2011). Development and preliminary testing of the new five-level version of EQ-5D (EQ-5D-5L). Qual Life Res.

[16] Dyer MTD, Goldsmith KA, Sharples LS (2010). A review of health utilities using the EQ-5D in studies of cardiovascular disease. Health Qual Life Outcomes.

[17] Luo N, Wang P, Thumboo J (2014). Valuation of EQ-5D-3L health states in Singapore: modeling of time trade-off values for 80 empirically observed health states. Pharmacoeconomics.

[18] Cohen J (1988). Statistical power analysis for the behavioral sciences.

[19] Machin D, Campbell M, Tan BT, Tan SH (2009). Sample size tables for clinical studies.

[20] Goh KT, Cutter J, Heng BH (2006). Epidemiology and control of SARS in Singapore. Ann Acad Med Singap.

[21] Yang Y, Li W, Zhang Q, Zhang L, Cheung T, Xiang YT (2020). Mental health services for older adults in China during the COVID-19 outbreak. Lancet Psychiatry.

[22] Zhang WR, Wang K, Yin L (2020). Mental health and psychosocial problems of medical health workers during the COVID-19 epidemic in China. Psychother Psychosom.

[23] Lai J, Ma S, Wang Y (2020). Factors associated with mental health outcomes among health care workers exposed to coronavirus disease 2019. JAMA Netw Open.

[24] Mazza C, Ricci E, Biondi S (2020). A nationwide survey of psychological distress among Italian people during the COVID-19 pandemic: immediate psychological responses and associated factors. Int J Environ Res Public Health.

[25] Gao J, Zheng P, Jia Y (2020). Mental health problems and social media exposure during COVID-19 outbreak. PLoS ONE.

[26] Tsai J, Wilson M (2020). COVID-19: a potential public health problem for homeless populations. Lancet Public Health.

[27] Liem A, Wang C, Wariyanti Y (2020). The neglected health of international migrant workers in the COVID-19 epidemic. Lancet Psychiatry.

[28] Hays RD, Kim S, Spritzer KL (2009). Effects of mode and order of administration on generic health-related quality of life scores. Value Health.

