# Supplementary material for: Impact of COVID-19 on health-related quality of life in patients with cardiovascular disease: a multi-ethnic Asian study
Source: Health Qual Life Outcomes. 2020 Dec 14;18:387. doi: 10.1186/s12955-020-01640-5 (PMC7734458; doi:10.1186/s12955-020-01640-5)
Supplement: Supplementary file 1 — Additional file 1. Supplemental Tables S1 and S2. [file 12955_2020_1640_MOESM1_ESM.docx]

**SUPPLEMENTAL MATERIAL**

Title: Impact of COVID-19 on health-related quality of life in patients with cardiovascular disease: a multi-ethnic Asian study

Shir Lynn Lim^1,2^, Kai Lee Woo^1^, Eleanor Lim^1^, Faclin Ng^1^, Mark YY Chan^1,2^, Mihir Gandhi^3,4,5^

Affiliations:

^1^Department of Cardiology, National University Heart Center, Singapore

^2^Department of Medicine, Yong Loo Lin School of Medicine, Singapore

^3^Biostatistic, Singapore Clinical Research Institute, Singapore

^4^Centre for Quantitative Medicine, Duke-NUS Medical School, Singapore

^5^Global Health Group, Center for Child Health Research, Tampere University, Finland

Corresponding author:

Shir Lynn Lim

National University Heart Center Singapore

Email: [shir_lynn_lim@nuhs.edu.sg](mailto:shir_lynn_lim@nuhs.edu.sg)

**Supplementary Table 1. Comparison of characteristics at pre-pandemic visit for patients who completed EQ-5D-3L and EQ-5D-5L with those who completed only EQ-5D-3L**

| Characteristics | EQ-5D-3L + EQ-5D-5L  cohort  N = 66 | EQ-5D-3L only cohort  N = 15 | P-value |
| --- | --- | --- | --- |
| Age (years), mean (SD) | 50.6 (10.4) | 56.2 (10.4) | 0.147 |
| ≤60 | 31 (47.0) | 10 (66.7) |  |
| >60 | 35 (53.0) | 5 (33.3) |  |
| Male, n (%) | 62 (93.9) | 13 (86.7) | 0.307 |
| Ethnicity, n (%) |  |  | 0.739 |
| Chinese | 49 (74.2) | 13 (86.7) |  |
| Malay | 10 (15.2) | 2 (13.3) |  |
| Indian | 6 (9.1) | 0 |  |
| Others | 1 (1.5) | 0 |  |
| Education, n (%) |  |  | 0.271 |
| Primary (6 years) or les | 12 (18.2) | 1 (6.7) |  |
| Secondary (up to 11 years) | 30 (45.5) | 5 (33.3) |  |
| Diploma, university or higher | 24 (36.4) | 9 (60.0) |  |
| Household earning <S$4000, n (%) | 34 (51.5) | 8 (53.3) | >0.999 |
| Heart problems, n (%) |  |  |  |
| Coronary artery disease | 41 (62.1) | 15 (100.0) | 0.004 |
| Heart failure | 22 (33.3) | 1 (6.7) | 0.055 |
| Arrhythmia | 12 (18.2) | 0 (0.0) | 0.110 |
| Other heart problems | 6 (9.1) | 0 (0.0) | 0.587 |
| Comorbidities, n (%) |  |  |  |
| Hypertension | 41 (62.1) | 9 (60.0) | >0.999 |
| Hyperlipidaemia | 42 (63.6) | 7 (46.7) | 0.253 |
| Diabetes | 38 (57.6) | 2 (13.3) | 0.003 |
| Stroke | 8 (12.2) | 1 (6.7) | >0.999 |
| Other | 37 (56.1) | 3 (20.0) | 0.020 |
| NYHA functional classification, n (%) |  |  | 0.004 |
| I | 31 (47.0) | 14 (93.3) |  |
| II | 30 (45.5) | 1 (6.7) |  |
| III | 5 (7.6) | 0 (0.0) |  |
| Health status using EQ-VAS, mean (SD) | 77.8 (12.7) | 82.1 (10.3) | 0.234 |
| EQ-5D-3L utility score, mean (SD) | 0.886 (0.20) | 0.952 (0.15) | 0.239 |
| EQ-5D-5L utility score, mean (SD) | 0.875 (0.18) | - |  |

EQ-5D-3L, 3-level EQ-5D; EQ-5D-5L, 5-level EQ-5D; SD, standard deviation; NYHA, New York Heart Association; EQ-VAS, EQ - Visual Analogue Scale.

**Supplementary Table 2. Comparison of EQ-5D-5L responses and dimension scores at pre- and during pandemic visits (N = 66)**

| Dimension  Response level, n (%) | Pre-pandemic |  | During pandemic |  |
| --- | --- | --- | --- | --- |
|  |  |  |  | P-value |
| Mobility |  |  |  | 0.180 |
| No problems | 57 (86.4) |  | 57 (86.4) |  |
| Slight problems | 8 (12.1) |  | 3 (4.6) |  |
| Some problems | 1 (1.5) |  | 6 (9.1) |  |
| Severe problems | 0 |  | 0 |  |
| Extreme problems | 0 |  | 0 |  |
| Mean dimension score (SD) | 1.152 (0.40) |  | 1.227 (0.60) |  |
| Mean change in dimension score (95% CI) | 0.076 (-0.056, 0.207) | | | 0.254 |
| Standardized effect size | 0.190 | | |  |
| Self-care |  |  |  | 0.999 |
| No problems | 64 (97.0) |  | 64 (97.0) |  |
| Slight problems | 2 (3.0) |  | 2 (3.0) |  |
| Some problems | 0 |  | 0 |  |
| Severe problems | 0 |  | 0 |  |
| Extreme problems | 0 |  | 0 |  |
| Mean dimension score (SD) | 1.030 (0.17) |  | 1.030 (0.17) |  |
| Mean change in dimension score (95% CI) | 0.000 (-0.061, 0.061) | | | 0.999 |
| Standardized effect size | 0.000 | | |  |
| Usual activities |  |  |  | 0.613 |
| No problems | 57 (86.4) |  | 57 (86.4) |  |
| Slight problems | 9 (13.6) |  | 6 (9.1) |  |
| Some problems | 0 |  | 3 (4.6) |  |
| Severe problems | 0 |  | 0 |  |
| Extreme problems | 0 |  | 0 |  |
| Mean dimension score (SD) | 1.136 (0.35) |  | 1.182 (0.49) |  |
| Mean change in dimension score (95% CI) | 0.045 (-0.080, 0.171) | | | 0.471 |
| Standardized effect size | 0.129 | | |  |
| Pain/discomfort |  |  |  | 0.199 |
| No problems | 40 (60.6) |  | 42 (63.6) |  |
| Slight problems | 23 (34.9) |  | 13 (19.7) |  |
| Moderate problems | 3 (4.6) |  | 8 (12.1) |  |
| Severe problems | 0 |  | 2 (3.0) |  |
| Extreme problem | 0 |  | 1 (1.5) |  |
| Mean dimension score (SD) | 1.439 (0.59) |  | 1.591 (0.93) |  |
| Mean change in dimension score (95% CI) | 0.152 (-0.082, 0.385) | | | 0.199 |
| Standardized effect size | 0.258 | | |  |
| Anxiety/depression |  |  |  | 0.0499 |
| No problems | 52 (78.8) |  | 44 (66.7) |  |
| Slight problems | 12 (18.2) |  | 12 (18.2) |  |
| Moderate problems | 2 (3.0) |  | 9 (13.6) |  |
| Severe problems | 0 |  | 1 (1.5) |  |
| Extreme problems | 0 |  | 0 |  |
| Mean dimension score (SD) | 1.242 (0.50) |  | 1.500 (0.79) |  |
| Mean change in dimension score (95% CI) | 0.258 (0.073, 0.442) | | | 0.007 |
| Standardized effect size | 0.516 | | |  |

Response levels were compared between two visits using the McNemar-Bowker exact test of symmetry.

Dimension score was compared between two visits using the paired t-test.

EQ-5D-5L, 5-level EQ-5D; SD, standard deviation; CI, confidence interval.
